# Dissecting the Effects of Periplasmic Chaperones on the *In Vitro* Folding of the Outer Membrane Protein PagP

**DOI:** 10.1016/j.jmb.2013.06.017

**Published:** 2013-09-09

**Authors:** Lindsay M. McMorran, Alice I. Bartlett, Gerard H.M. Huysmans, Sheena E. Radford, David J. Brockwell

**Affiliations:** Astbury Centre for Structural Molecular Biology, University of Leeds, Leeds LS2 9JT, UK; School of Molecular and Cellular Biology, University of Leeds, Leeds LS2 9JT, UK

**Keywords:** OMP, outer membrane protein, BAM, β-barrel assembly machine, OM, outer membrane, LPS, lipopolysaccharide, *di*C_12:0_PC, 1,2-dilauroyl-*sn*-glycero-3-phosphocholine, *di*C_12:0_PG, 1,2-dilauroyl-*sn*-glycero-3-phospho-(1′-*rac*-glycerol), LUV, large unilamellar vesicle, LPR, lipid-to-protein ratio, HT PagP, His-tagged PagP, membrane protein folding, PagP, Skp, SurA, folding kinetics

## Abstract

Although many periplasmic folding factors have been identified, the mechanisms by which they interact with unfolded outer membrane proteins (OMPs) to promote correct folding and membrane insertion remain poorly understood. Here, we have investigated the effect of two chaperones, Skp and SurA, on the folding kinetics of the OMP, PagP. Folding kinetics of PagP into both zwitterionic *di*C_12:0_PC (1,2-dilauroyl-*sn*-glycero-3-phosphocholine) liposomes and negatively charged 80:20 *di*C_12:0_PC:*di*C_12:0_PG [1,2-dilauroyl-*sn*-glycero-3-phospho-(1′-*rac*-glycerol)] liposomes were investigated using a combination of spectroscopic and SDS-PAGE assays. The results indicate that Skp modulates the observed rate of PagP folding in a manner that is dependent on the composition of the membrane and the ionic strength of the buffer used. These data suggest that electrostatic interactions play an important role in Skp-assisted substrate delivery to the membrane. In contrast, SurA showed no effect on the observed folding rates of PagP, consistent with the view that these chaperones act by distinct mechanisms in partially redundant parallel chaperone pathways that facilitate OMP assembly. In addition to delivery of the substrate protein to the membrane, the ability of Skp to prevent OMP aggregation was investigated. The results show that folding and membrane insertion of PagP can be restored, in part, by Skp in conditions that strongly favour PagP aggregation. These results illustrate the utility of *in vitro* systems for dissecting the complex folding environment encountered by OMPs in the periplasm and demonstrate the key role of Skp in holding aggregation-prone OMPs prior to their direct or indirect delivery to the membrane.

## Highlights

• An *in vitro* model was created to study chaperone-mediated PagP folding.
• SurA does not directly assist PagP folding into liposomes.
• Skp affects the rate of refolding in a manner dependent on liposome composition.
• Skp can prevent aggregation of PagP and promote folding into liposomes.
• Skp acts as a holdase and its interactions with lipid are electrostatically driven.

## Graphical abstract

**Figure:**
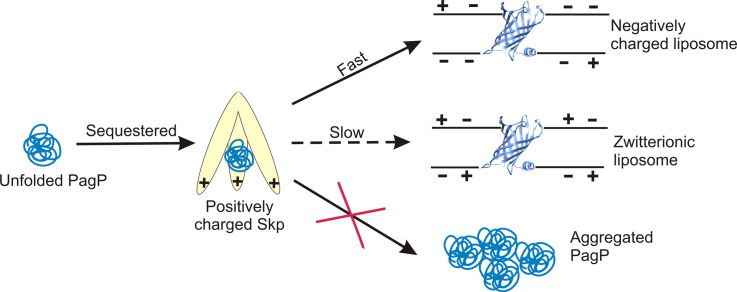

## Introduction

The *Escherichia coli* outer membrane (OM) is densely packed with outer membrane proteins (OMPs) that carry out a diverse range of functions that include (non)specific transport of small and large ligands [1–3], proteolytic and synthetic reactions [1,3] and cellular recognition and adhesion [2,3]. Despite their importance, understanding the biogenesis of OMPs is a formidable challenge. OMPs synthesised in the cytoplasm have an N-terminal signal sequence that targets them for translocation across the inner membrane by the SecYEG translocon [4]. Once in the periplasm, nascent OMPs may interact with a number of folding factors before reaching the OM. Finally, insertion of many OMPs into the OM is assisted by an assembly of proteins known as the β-barrel assembly machine (BAM) complex [5]. Reconstitution of the entire BAM complex *in vitro* demonstrated the importance of this machinery in assisting OMP assembly [6].

**Fig. 1:**
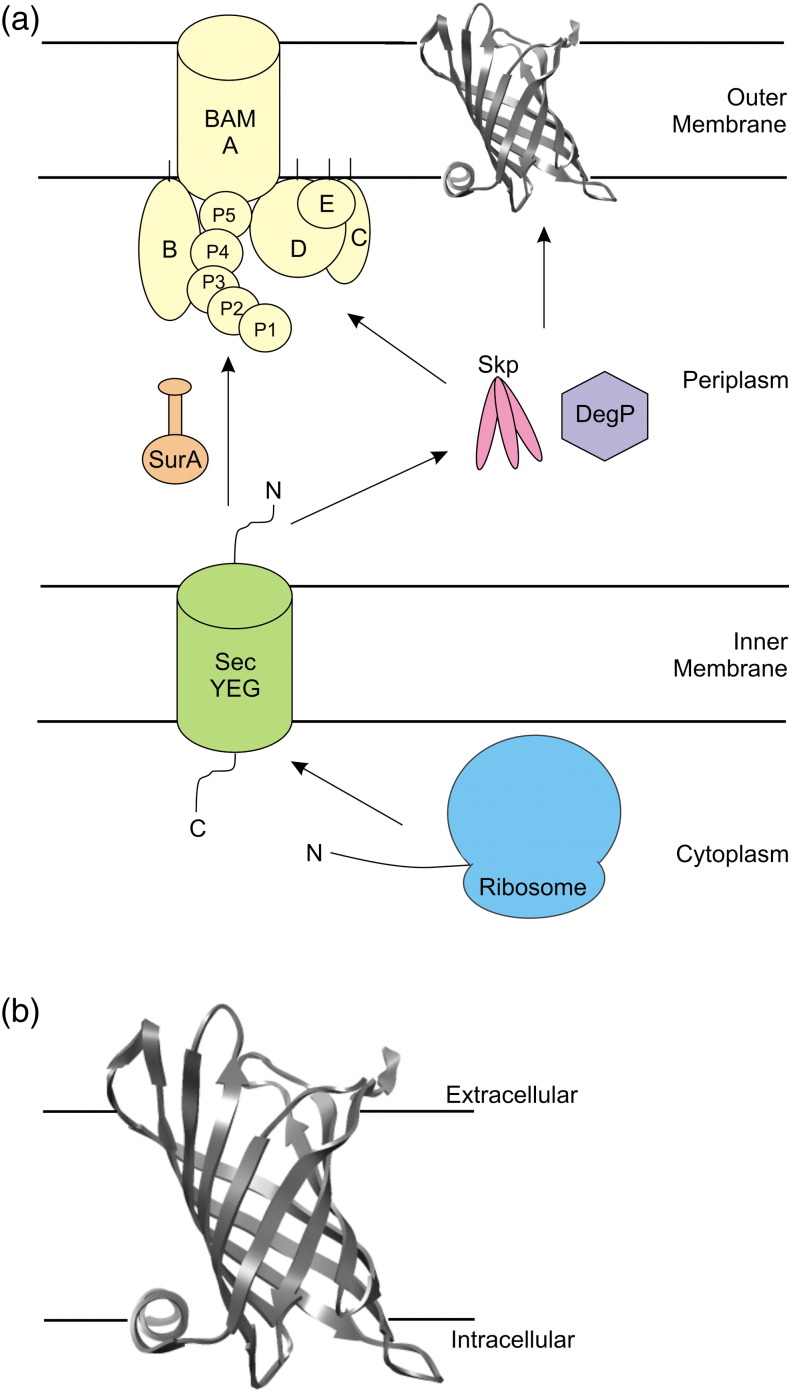
(a) Schematic of biogenesis of OMPs in *E. coli*: OMPs are synthesised on the ribosome before post-translational translocation across the inner membrane by the SecYEG translocon. Unfolded OMPs are then chaperoned across the periplasm to the β-barrel assembly (BAM) complex, which aids folding and insertion into the OM. BAM complex proteins are labelled A–E, and the periplasmic polypeptide transport-associated (POTRA) domains of BamA are labelled P1–5. (b) Crystal structure of PagP [11] (PDB: 1THQ) created using UCSF Chimera [12]. Black horizontal lines indicate the approximate position of the OM.

While numerous studies have demonstrated that the BAM complex is essential for the assembly of most OMPs *in vivo* [7], evidence from genetic studies has suggested that there is redundancy amongst some of the soluble periplasmic folding factors [8]. This has resulted in the proposal that parallel pathways exist for delivery of OMPs to the OM by molecular chaperones [8]. Sklar *et al.* and, more recently, Denoncin *et al*. suggested that SurA is the primary chaperone that assists in transport of OMPs across the periplasm to the OM, with DegP/Skp acting to rescue OMPs that deviate from this pathway [9,10] (Fig. 1a). Conversely, it has also been suggested that different OMPs may be preferentially bound by chaperones from one or other of these two pathways [13,14].

Previous studies have demonstrated that the trimeric chaperone, Skp, binds to bacterial OMPs maintaining the transmembrane domain in an unfolded conformation [15], by forming stable Skp:OMP complexes with dissociation constants in the nanomolar range [16]. SDS-PAGE analysis was used to demonstrate that Skp accelerates OmpA folding into negatively charged bilayers and impedes incorporation of OmpA into neutral bilayers [17] presumably due to the large macrodipole present on the chaperone surface [18]. The relationship of these observations to the role of Skp *in vivo*, however, remains unknown.

The periplasmic folding factor, SurA, contains two parvulin-like peptidylprolyl isomerase domains (P1 and P2) sandwiched between an N-terminal domain and smaller C-terminal domain [19]. Studies of SurA constructs from which the P1 and P2 domains had been removed demonstrated that SurA exhibits chaperone activity independent of peptidylprolyl isomerase activity [20]. SurA has been shown both to interact with BamA *in vivo* [9] and to assist BAM complex-dependent folding of OmpT *in vitro* [6,21], suggesting an important role of this chaperone in the OMP assembly pathway (Fig. 1a).

Here, we extend studies of chaperone-assisted folding of OMPs to include interactions with PagP, an OM acyl transferase enzyme from *E. coli*, which transfers a palmitate chain from a phospholipid to lipid A and hence reinforces the structure of the membrane [11]. PagP forms an eight-stranded antiparallel β-barrel with a 19-residue α-helix at its N-terminus [11,22,23] (Fig. 1b) and can fold to its native state *in vitro* in both detergent micelles and synthetic liposomes [24,25]. The *in vitro* folding pathway of PagP has been studied in detail, revealing a two-state, concerted folding and insertion mechanism *via* a polarised transition state [26], under defined folding and unfolding conditions [7–10 M urea, 50 mM sodium phosphate buffer, pH 8.0, and *di*C_12:0_PC (1,2-dilauroyl-*sn*-glycero-3-phosphocholine) liposomes]. In this pathway, folding and membrane insertion are initiated at the C-terminal region of the β-barrel, with the formation of the α-helix helping to lock the folded protein into the lipid bilayer [24,26]. The existence of parallel folding pathways has also been demonstrated [27], with the bilayer properties and the lipid-to-protein ratio (LPR) being important determinants of the route to the native state.

Here, we utilise direct spectroscopic measurements of the kinetics of folding and membrane insertion, as well as cold SDS-PAGE assays to analyse the population of folded PagP, to assess the role of Skp and SurA in the folding and insertion of a bacterial OMP into liposomes. Experiments that vary the lipid composition of the membrane and the ionic strength of the buffer are presented. The results reveal that Skp-mediated delivery of PagP to liposomes is influenced by electrostatic interactions between the chaperone and lipid. The ability of Skp to rescue proteins from aggregation and promote folding and membrane insertion is also demonstrated. Conversely, SurA does not appear to participate in direct membrane delivery of unfolded PagP. These data provide further evidence for multiple distinct pathways of chaperone-assisted transport of unfolded OMPs across the periplasm and insertion into the OM [9,28,29].

## Results

### Development of kinetic folding assays in low urea concentrations

Previous analysis of the PagP folding mechanism used a C-terminally His-tagged construct of PagP [the mature PagP followed by Leu, Glu and (His)_6_, referred to as His-tagged PagP (HT PagP) herein] that requires the presence of high concentrations of urea (> 4 M) to maintain the protein in a folding-competent state under the conditions employed (50 mM sodium phosphate, pH 8.0, in the presence of 100 nm *di*C_12:0_PC liposomes) [24,26,27]. Here, we use an untagged PagP construct (referred to as PagP) that has been shown previously to remain folding-competent in the presence of 1 M urea [25], facilitating biophysical studies of folding in the presence of periplasmic chaperones. HT PagP and PagP have markedly different stabilities in equilibrium unfolding experiments: the Leu-Glu-(His)_6_ sequence is destabilising (Fig. S1), resulting in a PagP construct that unfolds reversibly. By contrast, removal of the His-tag and linker sequence results in a more stable construct that shows hysteresis in unfolding (Fig. S1), a feature often observed in OMP folding studies [24,30,31]. Hysteresis in unfolding makes this construct unsuitable for equilibrium denaturation studies; however, its ability to remain folding-competent at low urea concentrations renders it suitable for kinetic folding assays in conjunction with water-soluble chaperones.

**Fig. 2:**
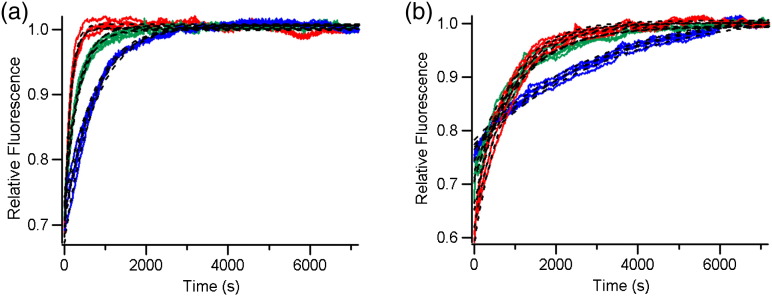
Example kinetic traces of PagP folding into liposomes using tryptophan fluorescence as a probe. Kinetic traces have been normalised to the final fluorescence signal to highlight the changes in *k*_obs_ in 2 M (blue lines), 3 M (green lines) and 4 M (red lines) urea for PagP folding into (a) *di*C_12:0_PC liposomes and (b) 80:20 *di*C_12:0_PC:*di*C_12:0_PG liposomes. Note that under these refolding conditions, a burst phase is observed in the dead time of the experiment. Fits to single exponential functions (after the burst phase) are shown as black broken lines. All kinetic samples contained 0.4 μM PagP, LPR 3200:1, and 50 mM glycine, pH 9.5, and were measured at 37 °C.

Analysis of folding kinetic transients of HT PagP into liposomes previously revealed the presence of a burst phase, most likely due to the adsorption of protein to the liposome, followed by a slower exponential phase, consistent with membrane insertion [26,27]. To characterise the folding of untagged PagP, we directly diluted an unfolded stock of PagP in 10 M urea into 100 nm *di*C_12:0_PC large unilamellar vesicles (LUVs) at an LPR of 3200:1 and we monitored folding using tryptophan (Trp) fluorescence as a probe. PagP has 12 Trp residues, 8 of which reside in the transmembrane region of the barrel, acting as sensitive probe of folding and membrane insertion. PagP folding was shown to be well described by a single exponential function in urea concentrations of ≥ 2 M (Fig. 2) over the time scale of the experiment. Interestingly, the observed rate of PagP folding increased fivefold (Table S1) with increasing urea concentration between 2 M and 4 M urea (Fig. 2a), suggesting that the presence of denaturant changes the balance between folding and aggregation to favour folding as the concentration of urea is increased. In agreement with this hypothesis, PagP has been shown to be particularly aggregation-prone compared with other OMPs [24,32] and fewer aggregates are observed in cold SDS-PAGE assays at higher urea concentrations (Fig. S2). The observed folding rate constants were independent of protein concentration between 0.04 and 0.4 μM (Table S2; Fig. S3). Folding of PagP into 80:20 *di*C_12:0_PC:*di*C_12:0_PG [1,2-dilauroyl-*sn*-glycero-3-phospho-(1′-*rac*-glycerol)] LUVs (Fig. 2b) was similarly well described by a single exponential function and, although the rate constants obtained were lower than those in 100% *di*C_12:0_PC (Table S1), the same trend of increasing rate constant with increasing urea concentration (fourfold increase between 2 M and 4 M urea) was observed.

In order to introduce soluble chaperones to the assay, an additional step was introduced in which the PagP solution was diluted from a high concentration (10 M) to a low concentration (0.24 M) of urea in the absence of lipid, either with or without chaperone. This was used to allow binding of the chaperone to PagP before the folding reaction was initiated by the addition of LUVs. Analytical gel filtration of PagP in 0.24 M urea yields a single peak, indicating that under these conditions, PagP populates a single unaggregated species (Fig. S4). Measuring PagP folding into *di*C_12:0_PC LUVs in the absence of chaperones after this initial dilution step revealed no change in the rate constant of folding relative to that for PagP in 10 M urea diluted directly into LUVs. For example, when PagP unfolded in 10 M urea is added to *di*C_12:0_PC LUVs at a final concentration of 2 M urea, the rate constant of folding is 1.34 × 10^− 3^ ± 7.7 × 10^− 5^ s^− 1^. The corresponding rate constant obtained when PagP is first incubated at 0.24 M urea for 5 min before addition to *di*C_12:0_PC LUVs at a final concentration of 2 M urea is 1.52 × 10^− 3^ ± 9.8 × 10^− 6^ s^− 1^, demonstrating that this construct remains folding-competent in these conditions. The ability to maintain PagP in a single unaggregated state in low concentrations of denaturant was surprising. This species may represent an early step in the folding pathway of PagP in liposomes and, as such, it was further characterised by spectroscopic methods.

**Fig. 3:**
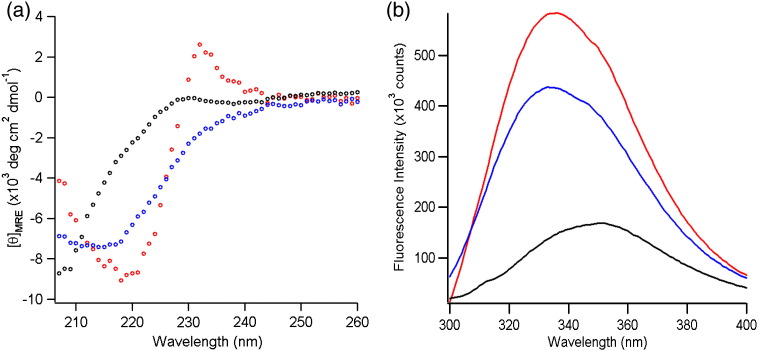
PagP refolds to its native state as assessed by far UV-CD and Trp fluorescence emission spectroscopy. (a) The far-UV CD spectra of 10 μM PagP refolded in 50 mM glycine, pH 9.5, in *di*C_12:0_PC liposomes (red circles); 10 μM PagP in 0.24 M urea and 50 mM glycine, pH 9.5 (blue circles); and 10 μM PagP unfolded in 10 M urea and 50 mM glycine, pH 9.5 (black circles). (b) The fluorescence emission spectra of 0.4 μM PagP folded in *di*C_12:0_PC liposomes and 50 mM glycine, pH 9.5 (red line); 0.4 μM PagP in 0.24 M urea and 50 mM glycine, pH 9.5 (blue line); and 0.4 μM PagP unfolded in 10 M urea and 50 mM glycine, pH 9.5 (black line). The 〈λ_320–370nm_〉 calculated for the unfolded spectrum in 10 M urea is 346.3 nm. The 〈λ_320–370nm_〉 values for the folded spectrum and spectrum in 0.24 M urea are 342.9 nm. All spectra were acquired at 37 °C. Corresponding data for HT PagP can be found in Fig. 3 of Huysmans *et al.*[24].

The far-UV CD spectrum of PagP in 0.24 M urea is distinct from that obtained after the folding reaction has reached completion (i.e., PagP folded and inserted into liposomes) and that of unfolded PagP (Fig. 3a). The far-UV CD spectrum of native PagP (Fig. 3a) shows the presence of a negative peak at 218 nm, indicative of β-sheet structure, and also a Cotton band at 232 nm, which has previously been shown to arise from close packing of Tyr26 and Trp66, a highly characteristic signature of native PagP [24,33]. This spectral feature is missing from the folding-competent state in 0.24 M urea, but β-sheet structure is indicated by negative ellipticity at 210–215 nm (Fig. 3a). By contrast with these results, the Trp fluorescence emission spectra of the folding-competent state and PagP after the folding reaction had reached completion showed identical λ_max_ values, but differed in intensity. Both spectra were blue shifted and the fluorescence yield was increased compared with the unfolded spectrum in 10 M urea (Fig. 3b). These observations show that in a low concentration of denaturant, the kinetic assays described above follow PagP folding from a water-soluble partially folded state to its native state.

### Addition of SurA does not change the observed rate of PagP folding

To assess the stability of SurA, we carried out equilibrium denaturation experiments, monitored using intrinsic Trp fluorescence. The resulting denaturation profile indicates that SurA remains natively folded in PagP folding assays containing 2 or 3 M urea (Fig. S5). Consequently, unfolded PagP in 10 M urea was diluted into a threefold molar excess of SurA in 50 mM glycine, pH 9.5, containing 0.24 M residual urea and incubated for 5 min. The sample was then diluted into liposomes at a final concentration of 2 or 3 M urea to facilitate folding and membrane insertion. The folding kinetics into *di*C_12:0_PC and 80:20 *di*C_12:0_PC:*di*C_12:0_PG LUVs were then measured using Trp fluorescence (see Materials and Methods).

**Fig. 4:**
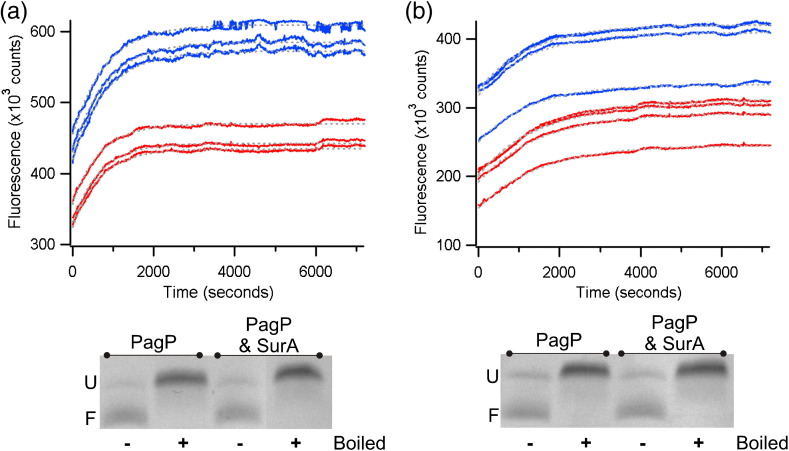
Kinetic traces for PagP folding into (a) *di*C_12:0_PC and (b) 80:20 *di*C_12:0_PC:*di*C_12:0_PG liposomes in the presence (blue lines) or absence (red lines) of a threefold molar excess of SurA. Grey broken lines represent the fits of the data to a single exponential function. Three replicate samples of each assay are shown. The differences in the final fluorescence signal arise from very small PagP concentration differences between samples, which appear due to the large molar extinction coefficient of PagP (see Materials and Methods). Note, however, that each sample refolded successfully with a similar folding yield. The lower panels show cold SDS-PAGE analysis of PagP samples allowed to fold overnight in the presence of a sixfold molar excess of SurA. The folded and unfolded forms of PagP are denoted by F and U, respectively. Kinetic samples contained 0.4 μM PagP and 2–3 M urea. SDS-PAGE gel samples contained 4 μM PagP and 1 M urea. All samples had an LPR of 3200:1, contained 50 mM glycine, pH 9.5, and were measured at 37 °C.

Inclusion of SurA in the refolding solution was found to have no effect on the observed rate constant of PagP folding into either zwitterionic *di*C_12:0_PC LUVs (*k*_obs_ = 1.34 × 10^− 3^ ± 4.5 × 10^− 5^ s^− 1^ for PagP alone and *k*_obs_ = 1.41 × 10^− 3^ ± 1.3 × 10^− 4^ s^− 1^ with SurA in buffer containing 2 M urea) or negatively charged mixed 80:20 *di*C_12:0_PC:*di*C_12:0_PG LUVs (*k*_obs_ = 7.9 × 10^− 4^ ± 1.4 × 10^− 4^ s^− 1^ for PagP alone and *k*_obs_ = 6.3 × 10^− 4^ ± 1.7 × 10^− 4^ s^− 1^ with SurA) (Fig. 4a and b, top). Analysis by cold SDS-PAGE at the endpoint of the folding reaction showed no change in folding yield when SurA was included in the assay (Fig. 4a and b, bottom). These results indicate that the presence of SurA has no effect on the yield or rate of PagP folding into liposomes that carry different net charges.

**Fig. 5:**
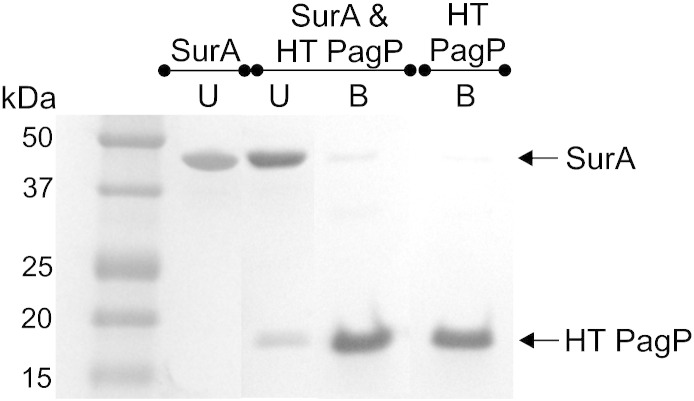
SurA does not bind to HT PagP under the conditions used for the PagP folding kinetic assays. HT PagP (10 μM) was immobilised on nickel Sepharose resin before incubation with 10 μM SurA in 50 mM glycine buffer, pH 9.5. SurA remains in the unbound (U) fraction, while HT PagP is bound (B) to the resin until eluted with 500 mM imidazole. Control experiments containing HT PagP or SurA only were conducted under identical conditions for comparison. Bound and unbound fractions were analysed by SDS-PAGE.

To assess whether PagP interacts with SurA in the absence of lipid, HT PagP was immobilised on to nickel Sepharose resin and incubated with an equal concentration of SurA at pH 9.5. Analysis of the unbound fraction indicated that SurA remains in solution, providing no evidence of an interaction between the two proteins (Fig. 5). Additionally, analytical gel filtration of SurA and PagP in solution showed no evidence of interaction between these two proteins in the absence of lipid (Fig. S6).

### The presence of Skp has a dramatic effect on observed rates of PagP folding

Prior to assessing the effect of Skp on PagP folding, the stability of Skp to chemical denaturants was measured using far-UV CD spectroscopy (Fig. S7). In 50 mM glycine, pH 9.5, at 37 °C, the midpoint of the Skp equilibrium denaturation curve ([urea]_50%_) occurs at ~ 1.4 M urea. Kinetic folding assays in the presence of Skp were carried out using the same method as that described for SurA; however, a final concentration of 2 M urea was used for all experiments (see Materials and Methods). Trimeric Skp has been shown to bind to OMPs with a stoichiometry of one trimer per OMP monomer [16,34]. To ensure that an excess of Skp trimers were available to bind PagP, we added Skp into the PagP kinetic assay at a twofold molar excess of Skp trimers.

**Fig. 6:**
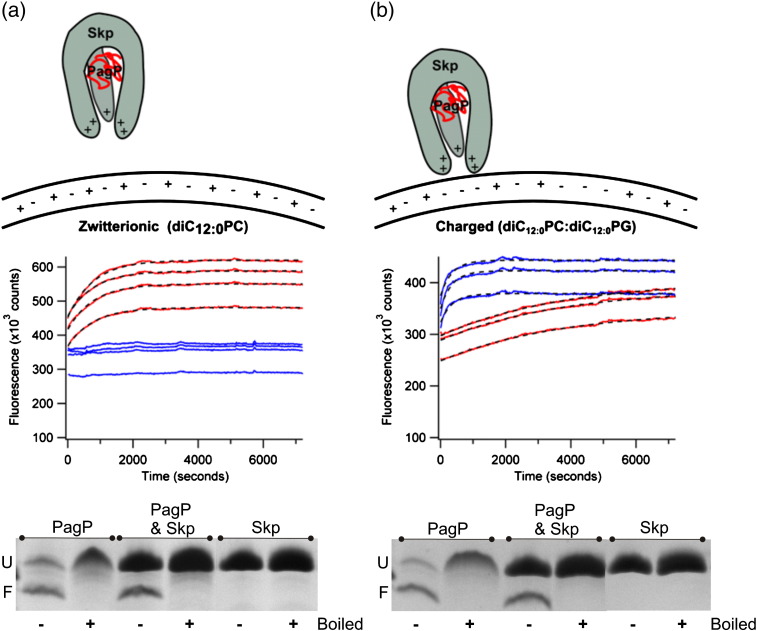
Kinetic traces of PagP folding into (a) *di*C_12:0_PC and (b) 80:20 *di*C_12:0_PC:*di*C_12:0_PG liposomes in the presence (blue lines) or absence (red lines) of a twofold molar excess of Skp trimers. Black broken lines represent the fits of the data to a single exponential function. Four replicate samples of each assay are shown. The differences in the final fluorescence signal arise from very small PagP concentration differences between samples, which appear due to the large molar extinction coefficient of PagP (see Materials and Methods). Note, however, that each sample refolded successfully with a similar folding yield. The lower panels show cold SDS-PAGE analysis of PagP samples allowed to fold overnight in the presence of a twofold molar excess of Skp trimers. The folded and unfolded forms of PagP are denoted by F and U, respectively. Kinetic samples contained 0.4 μM PagP and 2 M urea in 50 mM glycine, pH 9.5. SDS-PAGE gel samples contained 4 μM PagP and 1 M urea in 50 mM glycine, pH 9.5. All samples had an LPR of 3200:1 and were measured at 37 °C.

**Fig. 7:**
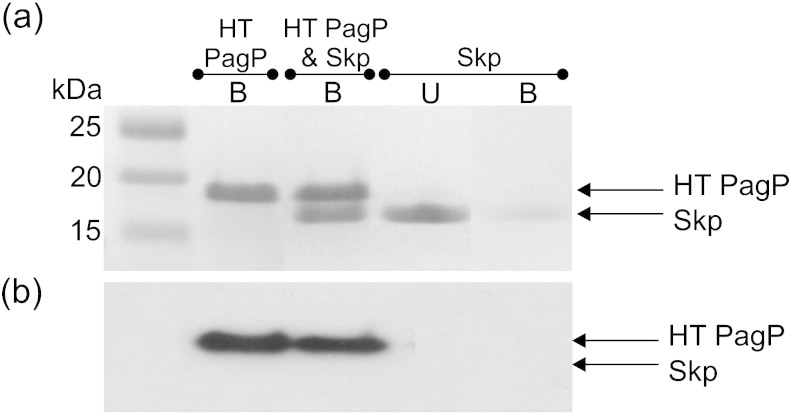
Skp binds to HT PagP under the conditions used for the PagP folding kinetic assays as analysed by (a) SDS-PAGE and (b) Western blot analysis with an anti-His-tag antibody. HT PagP (10 μM) was immobilised on nickel Sepharose resin before incubation with 30 μM Skp in 50 mM glycine buffer, pH 9.5. Control experiments containing HT PagP or Skp only were conducted under identical conditions for comparison. Skp alone does not bind to the resin and is present in the unbound (U) fraction, while in the presence of HT PagP, Skp co-elutes in the bound (B) fraction.

The results of experiments to monitor the effect of Skp trimers on PagP folding into *di*C_12:0_PC or 80:20 *di*C_12:0_PC:*di*C_12:0_PG liposomes (Fig. 6) show that Skp has a dramatic impact on the observed rate of PagP folding that is dependent on liposome composition. The presence of Skp slows folding into zwitterionic *di*C_12:0_PC LUVs to the extent that no folding is observed during a 2-h fluorescence time course (Fig. 6a, top and middle; Fig. S8). Cold SDS-PAGE analysis of samples that had been allowed to fold overnight (16 h, 37 °C), however, indicates that Skp does not affect the final yield of folded PagP under these conditions (Fig. 6a, bottom). By contrast, when PagP was folded into LUVs with a net negative charge (80:20 *di*C_12:0_PC:*di*C_12:0_PG), Skp increased the observed rate constant of PagP folding almost ninefold (*k*_obs_ PagP folding alone = 2.8 × 10^− 4^ ± 1.8 × 10^− 5^ s^− 1^, *k*_obs_ PagP folding in the presence of Skp = 2.4 × 10^− 3^ ± 4.3 × 10^− 4^ s^− 1^ for folding into buffer containing 2 M urea) (Fig. 6b, middle; Table S3). Despite this order of magnitude increase in apparent rate constant, SDS-PAGE assays indicated that the presence of Skp did not change the yield of folded PagP (Fig. 6b, bottom). As Skp is a basic protein (p*I* ~ 9.5), these results point to electrostatic interactions playing a major role in modulating the Skp-mediated delivery of PagP to the membrane, as suggested previously [18]. Skp and PagP have been shown recently to form a strong complex (*K*_d_ = 11.8 nM at pH 8) [35]. To confirm this observation under the experimental conditions used here, we incubated nickel Sepharose-immobilised HT PagP with an equal concentration of Skp trimers. In contrast with the results obtained for SurA, Skp was found to bind to the resin and co-elute with HT PagP upon addition of 500 mM imidazole (Fig. 7a and b).

### Electrostatic interactions are partially responsible for the effect of Skp on the observed rate of PagP folding into the bilayer

**Fig. 8:**
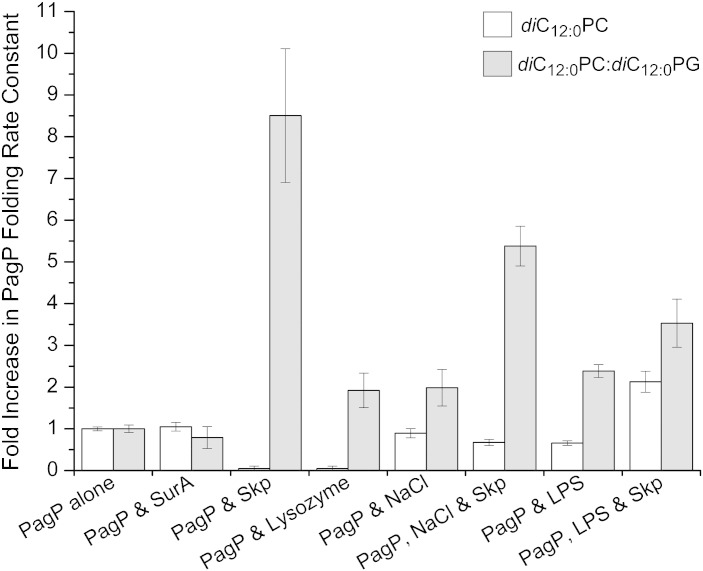
Relative rates of PagP folding under different conditions in *di*C_12:0_PC (white) and 80:20 *di*C_12:0_PC:*di*C_12:0_PG (grey) liposomes. Each rate is normalised to the rate of PagP folding alone in the same lipid. Error bars depict the standard deviation of the average rate for each condition, propagated through the normalisation. All kinetic samples contained 0.4 μM PagP, LPR of 3200:1, and 50 mM glycine, pH 9.5, and were measured at 37 °C. Unnormalised rate constants and their associated errors are provided in Table S3.

To investigate further the role of electrostatic interactions in modulating delivery of PagP to *di*C_12:0_PC and 80:20 *di*C_12:0_PC:*di*C_12:0_PG liposomes, we measured the effects of including another basic protein in the kinetic assays. Hen egg white lysozyme, which has the same p*I* as Skp, was added in a threefold molar excess to assays examining PagP folding into both *di*C_12:0_PC and mixed 80:20 *di*C_12:0_PC:*di*C_12:0_PG LUVs (Fig. 8; Table S3). In *di*C_12:0_PC LUVs, lysozyme showed a similar inhibition of PagP folding over the 2-h fluorescence time course to that of PagP incubated with an excess of Skp trimers (data not shown). Inclusion of lysozyme in 80:20 *di*C_12:0_PC:*di*C_12:0_PG LUVs gave rise to a small (~ 2-fold) increase in observed rate constant for PagP folding (*k*_obs_ = 7.9 × 10^− 4^ ± 1.4 × 10^− 4^ s^− 1^ for PagP alone and *k*_obs_ = 1.5 × 10^− 3^ ± 1.8 × 10^− 4^ s^− 1^ with lysozyme, data not shown). This contrasts with the dramatic 10-fold increase in the rate constant of folding into these liposomes in the presence of Skp. Analytical gel-filtration experiments suggest that lysozyme interacts with PagP in the absence of lipid (Fig. S9a). Similarly to the results for Skp, lysozyme was observed to co-elute from Ni-Sepharose resin with HT PagP (Fig. S9b).

These experiments demonstrate that electrostatic interactions can differentially modulate the rate of PagP folding into bilayers of different net charge. Such interactions could play an important role in substrate recognition and membrane delivery by the chaperone Skp. In the presence of zwitterionic bilayers, both lysozyme and Skp can retard PagP folding. Binding of both lysozyme and Skp to PagP has been demonstrated (Fig. 7; Fig. S9), but neither lysozyme nor Skp promotes the rate of folding of PagP under these conditions, most likely as there are no favourable interactions with the *di*C_12:0_PC LUVs. By contrast, both lysozyme and Skp can accelerate PagP folding into negatively charged 80:20 *di*C_12:0_PC:*di*C_12:0_PG LUVs. Favourable electrostatic interactions between lysozyme and the membrane presumably bring PagP closer to the lipid surface, promoting lipid adhesion and subsequent folding. Addition of Skp showed a dramatic relative acceleration of the PagP folding rate constant (~ 9-fold) in comparison with the acceleration induced by lysozyme (~ 2-fold), which may arise due to the structure and electrostatic surface pattern of trimeric Skp and its interactions with both substrate and lipid. Sequestration of the unfolded PagP into the central hydrophobic cavity of Skp [36], together with the complementary electrostatic interactions between the “tips” of the Skp trimer [36] and the negatively charged membrane surface, would serve to orient the unfolded PagP directly on to the membrane surface, greatly increasing the rate of folding. These data indicate, therefore, that the increased *k*_obs_ of PagP folding in the presence of Skp is not due to Skp acting simply as a non-specific electrostatic delivery system (see Discussion).

To investigate whether changes in the rate constant of PagP folding in the presence of Skp require complementary electrostatic interactions, we performed kinetic assays in the presence of sodium chloride (NaCl). Inclusion of 200 mM NaCl in the folding buffer led to similar observed rate constants of PagP folding into *di*C_12:0_PC LUVs in the presence or absence of Skp (*k*_obs_ = 8.1 × 10^− 4^ ± 9.6 × 10^− 5^ s^− 1^ and *k*_obs_ = 1.2 × 10^− 3^ ± 1.4 × 10^− 4^ s^− 1^, respectively) (Fig. 8; Table S3), indicating that increasing the ionic strength ablates the inhibition of PagP folding when Skp is present. In 80:20 *di*C_12:0_PC:*di*C_12:0_PG LUVs, 100 mM NaCl was included in the buffer as the kinetic traces became too noisy to fit with confidence in the presence of higher concentrations of NaCl. The presence of salt has been reported previously to increase the aggregation propensity of OMPs *in vitro* [32], and this, coupled with the slow folding rate constants observed for PagP folding in 80:20 *di*C_12:0_PC:*di*C_12:0_PG liposomes, could account for the need to use lower salt concentrations to obtain reliable kinetic traces. Despite these difficulties, folding of PagP into 80:20 *di*C_12:0_PC:*di*C_12:0_PG liposomes in the presence of Skp resulted in an approximately threefold increase in folding rate constant (*k*_obs_ = 5.6 × 10^− 4^ ± 6.2 × 10^− 5^ s^− 1^ for PagP alone and *k*_obs_ = 1.5 × 10^− 3^ ± 9.4 × 10^− 5^ s^− 1^ with Skp both in the presence of 100 mM NaCl), compared with the ninefold increase in the folding rate observed in the absence of salt (Fig. 8; Table S3). These data and the relative effects of Skp and lysozyme suggest that Skp-accelerated folding of PagP is not driven solely by electrostatic interactions.

Cross-linking experiments [37] have indicated that Skp interacts with nascent OMPs soon after they are translocated into the periplasm. Under normal growth conditions, Skp is not thought to play a major role in the delivery of OMPs to the OM itself [7]. The presence of a putative lipopolysaccharide (LPS) binding site on Skp [36] and studies of OmpA folding in which LPS was shown to modulate Skp-assisted delivery of OmpA to the membrane [17,34,38] suggest, however, that Skp may play a role in the delivery to the OM under conditions of stress [7]. To test this hypothesis, we incorporated 20% (w/w) LPS into both *di*C_12:0_PC and 80:20 *di*C_12:0_PC:*di*C_12:0_PG liposomes and measured the rate constant of PagP refolding as described above in the absence or presence of a twofold molar excess of Skp trimers. While incorporation of LPS into *di*C_12:0_PC LUVs doubled the PagP folding rate constants in the presence of Skp (*k*_obs_ = 8.9 × 10^− 4^ ± 6.7 × 10^− 5^ s^− 1^ for PagP alone and *k*_obs_ = 1.9 × 10^− 3^ ± 3.3 × 10^− 4^ s^− 1^ with Skp), a smaller increase (~ 50%) in the folding rate constant was observed in 80:20 *di*C_12:0_PC:*di*C_12:0_PG LUVs (*k*_obs_ = 6.8 × 10^− 4^ ± 7.1 × 10^− 6^ s^− 1^ for PagP alone and *k*_obs_ = 1.0 × 10^− 3^ ± 1.5 × 10^− 4^ s^− 1^ with Skp) (Fig. 8; Table S3). These data suggest that LPS, a negatively charged glycolipid, is able to increase Skp-mediated PagP folding rates into membranes that are not already negatively charged. Furthermore, the increase in the Skp-mediated PagP folding rate constant is smaller than that observed into negatively charged liposomes in the absence of LPS, suggesting that the presence of LPS in the bilayer may impede delivery of PagP to the membrane by Skp. This observation suggests that any specific Skp:LPS interactions would not assist in Skp-mediated delivery of OMPs to LPS-containing membranes. LPS has been shown to be able to insert spontaneously into lipid bilayers from aqueous solution [39]. In this case, an electrostatic interaction between LPS and Skp would allow Skp to come into close contact with the bilayer as the LPS inserts. This in turn would bring the OMP into contact with the membrane and trigger faster folding, as reported for OmpA [34].

### Skp displays holdase activity against a highly aggregation-prone PagP construct

**Fig. 9:**
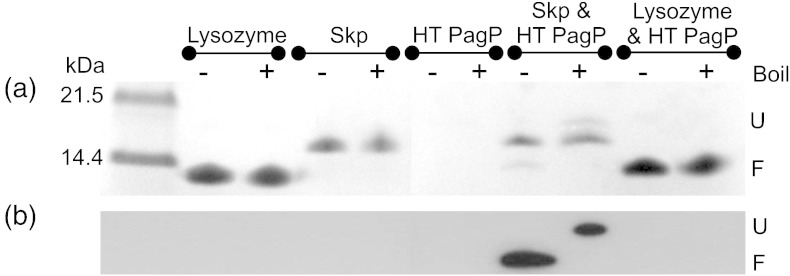
The holdase activity of Skp against HT PagP, a highly aggregation-prone construct, as demonstrated by (a) SDS-PAGE and (b) Western blot analysis using an anti-His-tag antibody. HT PagP (21.6 μM) in 1 M urea and 50 mM glycine, pH 9.5, was incubated in the presence of a twofold molar excess of Skp trimers or a sixfold molar excess of hen egg white lysozyme before a sixfold dilution into *di*C_12:0_PC liposomes (final HT PagP concentration = 3.6 μM; LPR 3200:1) at 37 °C. Any precipitate was removed by centrifugation and the supernatant was analysed for the presence of HT PagP. The folded and unfolded forms of HT PagP are denoted by F and U, respectively. Control experiments containing Skp and lysozyme incubated alone were also conducted under identical conditions. Lysozyme runs at the same apparent molecular weight as folded HT PagP. The ability of Skp to divert HT PagP from aggregation was quantified by densitometry measurements (Materials and Methods).

The data described above and elsewhere [35] show that Skp (or lysozyme) interacts with unfolded or partially folded PagP species. While this interaction slows folding into zwitterionic liposomes, it presumably decreases the local concentration of unfolded PagP in solution, decreasing aggregation. To test this idea further, we assessed the ability of Skp and lysozyme to prevent client protein aggregation using HT PagP [22,24] as this construct has been shown to require the presence of high concentrations of urea (> 4 M) in the buffer to remain folding-competent [24]. When unfolded HT PagP is diluted first into 1 M urea, then added to *di*C_12:0_PC LUVs 5 min later (see Materials and Methods), all of the HT PagP precipitated, leaving no protein detectable in the supernatant by either cold SDS-PAGE (Fig. 9a) or Western blot analysis using an anti His-tag antibody (Fig. 9b). By contrast, the presence of a twofold molar excess of Skp trimers in the 1 M urea solution was shown to prevent precipitation and to retain a significant proportion (12% and 14% for “cold” and heat-treated SDS-PAGE samples, respectively) of HT PagP in a folding-competent state as evidenced by the heat sensitivity of the HT PagP band in gel assays, which is characteristic of correctly folded OMPs [40] (Fig. 9). Incubation of HT PagP with a sixfold molar excess of lysozyme failed to prevent the precipitation of any of the HT PagP in the sample (Fig. 9). Taken together, the results suggest that Skp is able to sequester HT PagP, preventing its aggregation, and to then release the protein in a conformation that is able to fold spontaneously into *di*C_12:0_PC membranes. By contrast, lysozyme was unable to prevent aggregation of HT PagP or promote membrane insertion in this experiment. Consistent with other studies [15,17,41,42], these data support the view that Skp acts as a holdase, maintaining HT PagP in a folding-competent state in conditions that strongly favour aggregation.

## Discussion

Here, the development of spectroscopic assays to directly monitor the folding of PagP into liposomes in the presence of periplasmic folding factors has been described. We have used these assays to elucidate the role of individual periplasmic folding factors in assisting the folding of the OMP PagP. We show that the inclusion of SurA in folding assays had no effect on either rate or yield of PagP folding in either zwitterionic *di*C_12:0_PC or negatively charged 80:20 *di*C_12:0_PC:*di*C_12:0_PG liposomes, suggesting that SurA does not play a direct role in assisting folding *in vitro*. Accordingly, SurA is the only soluble periplasmic folding factor to have been cross-linked to BamA [9,43] and has been hypothesised to be involved in the major pathway for OMP transport across the periplasm *in vivo* [44]. Furthermore, in a recent study, the BAM complex was reconstituted *in vitro* and used to assist folding of OmpT into liposomes [6]. The presence of SurA was shown to increase the rate of OmpT folding by the BAM complex, as determined by monitoring the increase of enzymatic activity of OmpT over the course of the experiment [6]. While SurA has been shown to bind to peptides derived from OMP sequences [28,29], binding with affinities in the micromolar range [28], very few studies have reported binding of SurA to a full-length OMP *in vitro* [19,45]. Taken together with the results presented herein, the data suggest that SurA may be involved in the delivery of its substrates to the BAM complex, but in the absence of BAM, SurA does not affect the folding pathway of PagP into liposomes.

The inclusion of Skp in PagP folding assays demonstrated a dramatic impact of this chaperone on the rate of folding and membrane insertion, which is highly dependent on bilayer charge and the ionic strength of the buffer. Folding of PagP into zwitterionic *di*C_12:0_PC liposomes was inhibited such that no folding was observed over a 2-h time course. When the folding experiment was allowed to proceed overnight, no change in folded PagP yield was observed relative to that in the absence of Skp, suggesting that in the presence of liposomes, PagP sequestered by Skp is slowly released from the chaperone in a folding-competent state. In contrast with this behaviour, PagP folding into negatively charged 80:20 *di*C_12:0_PC:*di*C_12:0_PG liposomes was accelerated in the presence of Skp, suggesting that electrostatic interactions with the lipid head groups play an important role in Skp-mediated membrane delivery. Consistent with this, the crystal structure of the Skp trimer shows positively charged regions on the tip of each subunit of the protein [36] through which interaction with a negatively charged membrane would be favourable. The results obtained here using kinetic analysis of PagP folding accord with previous observations made using SDS-PAGE analysis to monitor OmpA folding in the presence of Skp [17]. In the latter case, Skp was shown to facilitate OmpA folding into negatively charged membranes at neutral or basic pH [17]. By contrast, Skp inhibited folding into neutral liposomes composed of 1,2-dioleoyl-*sn*-glycero-3-phosphocholine and the yield of folded OmpA at the end of the 4-h time course was reduced [17].

The importance of electrostatic interactions for Skp function was further demonstrated by the differential effects of NaCl on the folding rates of PagP into zwitterionic and negatively charged membranes and the ability of negatively charged LPS to accelerate Skp-assisted folding by a greater extent into zwitterionic relative to negatively charged membranes. The ability of lysozyme to partially mimic Skp also supports this idea. The decreased magnitude of its effect relative to Skp, however, indicates that other factors such as hydrophobic interactions and the conformation of the substrate and its orientation relative to the membrane also play a role.

If SurA is the chaperone involved in the major pathway of OMP biogenesis *in vivo*, what is the role of Skp? One attractive model is that Skp, in conjunction with DegP, binds to proteins that veer from the primary route, acting as a secondary, partially redundant pathway. The Skp trimer has been shown to have a central cavity enriched with hydrophobic residues [36], which has been suggested to be able to sequester unfolded OMPs. Indeed, a combination of NMR data and cross-linking studies revealed the ability of Skp to sequester the unfolded β-barrel domain of OmpA, while allowing the soluble periplasmic domain to remain outside the cavity and fold independently [15]. More recently, a study of OmpC, OmpF, and the transmembrane domain of OmpA used fluorescent labelling of Skp to show that all of these OMPs can enter the Skp cavity *via* the positively charged subunit tips, led by the N-terminus of the OMP [41]. The ability of Skp to bind a wide range of partially folded OMPs to sequester them from the complex periplasmic milieu is ideal for its proposed function *in vivo*. Here, by using a simplified *in vitro* system, we have shown that Skp can hold an extremely aggregation-prone OMP in solution and by doing so allow its quantitative folding and insertion into membranes.

## Materials and Methods

### Protein purification

Plasmid pET11a (Novagen) containing the mature form of PagP was kindly provided by Karen Fleming (Johns Hopkins University) [25] and plasmid pETCrcAHΔS containing HT PagP was kindly provided by Russell Bishop (McMaster University) [22]. Over-expression of both PagP constructs followed the method described by Burgess *et al*. [25]. Briefly, cell pellets were resuspended in 50 mM Tris–HCl and 5 mM ethylenediaminetetraacetic acid, pH 8.0, containing 1 mM PMSF and lysed by sonication. Inclusion bodies were pelleted by centrifugation (25,000***g***, 30 min, 4 °C). The pellet from 0.5 L of culture was resuspended in 20 ml of 50 mM Tris–HCl, pH 8.0, containing 2% (v/v) Triton X-100 and stirred at room temperature for 1 h to dissolve the membranes. The inclusion bodies were pelleted by centrifugation as before and resuspended in the detergent mixture a second time. The inclusion bodies were then pelleted by centrifugation and resuspended in 50 mM Tris–HCl, pH 8.0. The resuspended pellet was left stirring for 1 h at room temperature to ensure removal of residual detergent. The wash step in 50 mM Tris–HCl, pH 8.0, was repeated twice, pelleting the inclusion bodies by centrifugation after each resuspension.

To purify the protein further, we solubilised inclusion bodies in denaturing buffer (6 M GuHCl and 25 mM Tris–HCl, pH 8.0) and clarified them by centrifugation (20,000***g***, 20 min, 4 °C). The supernatant was filtered through a 0.2-μM syringe filter before loading on to a Superdex 75 HiLoad 26/60 gel-filtration column (GE Healthcare) mounted on an ÄKTA Prime chromatography system and equilibrated with two column volumes of the same buffer. Following gel filtration, PagP-containing fractions were concentrated to approximately 500 μM using Vivaspin 20 concentrators (molecular mass cutoff, 5 kDa; Sartorius). Purification of the HT PagP construct from inclusion bodies was carried out as previously described [24]. Purity was assessed by SDS-PAGE and protein concentration was quantified by UV absorbance (PagP ε_280_ = 82,390 M^− 1^ cm^− 1^, estimated using the ExPASy ProtParam tool [46]).

To over-express and purify Skp, we cultured *E. coli* BL21[DE3] cells (Stratagene) transformed with a pET21b plasmid encoding Skp (kindly provided by Jim Bardwell, University of Michigan) in LB medium at 27 °C, with shaking. Protein expression was induced at an OD_600_ (optical density at 600 nm) of ~ 0.6 by addition of sterile IPTG to give a final concentration of 25 μM. The cells were then grown overnight before harvesting. The cell pellet was resuspended in 20% sucrose (w/v) and 20 mM Tris–HCl, pH 8.0, and incubated on ice with 5 mM ethylenediaminetetraacetic acid and 0.1 mg ml^− 1^ lysozyme for 20 min. MgCl_2_ was added to give a final concentration of 20 mM and the spheroplasts were sedimented by centrifugation (12,000***g***, 20 min, 4 °C). The resulting periplasmic extract was dialysed against 20 mM Tris–HCl, pH 8.0, and 100 mM NaCl (Buffer A) overnight at 4 °C. The periplasmic extract was filtered and loaded onto a HiTrap Q (5 ml) column (GE Healthcare) and washed with 3 column volumes of Buffer A. The flow through from this column was then loaded onto a HiTrap SP (5 ml) column (GE Healthcare) and washed with 5 column volumes of buffer A, and Skp was eluted with a gradient running from 0% to 100% Buffer B (20 mM Tris–HCl, pH 8.0, and 750 mM NaCl) over 15 column volumes. Purified Skp was dialysed against 20 mM Tris–HCl, pH 8.0, to remove NaCl and concentrated to ~ 150 μM using Vivaspin 20 concentrators (molecular mass cutoff, 5 kDa; Sartorius). Purity was assessed by SDS-PAGE and protein concentration was quantified by UV absorbance (Skp ε_280_ = 1490 M^− 1^ cm^− 1^, estimated using the ExPASy ProtParam tool [46]).

Plasmid pET28b encoding the mature form of SurA with an N-terminal His-tag was kindly provided by Daniel Kahne (Harvard University) [6,21]. Over-expression and purification of SurA followed the method of Hagan *et al*. [6]. The N-terminal His-tag was removed using a CleanCleave kit (Sigma), following the manufacturer's recommended protocol and any residual His-tagged SurA removed using a Ni-Sepharose (GE Healthcare) pull down. The cleaved SurA was concentrated to ~ 150 μM using Vivaspin 20 concentrators (molecular mass cutoff, 5 kDa; Sartorius) and then diluted fivefold with Tris-buffered saline (pH 7.4) to remove residual imidazole, before concentrating again to ~ 150 μM. Purity was assessed by SDS-PAGE and protein concentration was quantified by UV absorbance (SurA ε_280_ = 29,450 M^− 1^ cm^− 1^ estimated using the ExPASy ProtParam tool [46]).

### Preparation of liposomes

The following lipids were used in PagP folding assays: *di*C_12:0_PC, *di*C_12:0_PG and LPS. *di*C_12:0_PC and *di*C_12:0_PG were purchased from Avanti Polar Lipids (Alabama, USA) (distributed in Europe by Instruchemie, Delfzijl, The Netherlands). LPS from *E. coli* strain 0111:B4 was purchased from Sigma (UK). Appropriate mixtures of lipids were dissolved in 90:10 (v/v) chloroform:methanol in glass test tubes. Solvent was then removed by drying under a gentle stream of N_2_, followed by further drying *in vacuo* for > 2.5 h. The resulting thin lipid film was resuspended in buffer to give a 40 mM lipid solution and left to stand at room temperature for 30 min. LUVs (100 nm) were prepared by extruding the lipid mixtures 11 times through 0.1 μM polycarbonate membranes (Nuclepore, Whatman, Clifton, NJ) using a mini extruder (Avanti, Alabaster, AL).

### Far-UV CD

Far-UV CD spectra were acquired on a Chirascan plus circular dichroism spectrometer (Applied PhotoPhysics) with a bandwidth of 1 nm, a scan speed of 20 nm min^− 1^, a step size of 1 nm and a path length of 0.1 mm. The average of eight scans was taken to enhance the signal-to-noise ratio. Folded PagP samples were prepared in 4 M urea and unfolded PagP samples contained 10 M urea. All samples contained 50 mM glycine buffer, pH 9.5, 37 °C, at a protein concentration of 10 μM. An LPR of 800:1 was used to reduce light scattering. Samples were refolded for 16 h before measurement. Corresponding blank spectra were subtracted for each sample.

### Analytical gel filtration

A stock solution of PagP unfolded in 50 mM glycine, pH 9.5, in 10 M urea was diluted to 10 μM in 50 mM glycine, pH 9.5, in 0.24 M urea and allowed to incubate at room temperature for 5 min in the presence of 60 μM SurA to analyse the interaction between PagP and SurA. PagP (2 μM) was added to 6 μM lysozyme in 50 mM glycine, pH 9.5, containing 0.24 M urea and incubated for 5 min at room temperature to analyse the interaction between lysozyme and PagP. In all experiments, 200 μL of the sample was then injected onto a Superdex 75 10/300 GL column (GE Healthcare, UK) equilibrated in 50 mM glycine, pH 9.5, and controlled by an ÄKTA prime purification system. The protein was eluted at a flow rate of 0.5 ml/min and 0.5-ml fractions were collected for analysis by SDS-PAGE. Under these conditions, lysozyme interacted with the Superdex resin, hence the requirement to use very low protein concentrations, which prevented the analysis of the eluted fractions by SDS-PAGE. Appropriate PagP-only, SurA-only and lysozyme-only samples were also analysed.

### Nickel sepharose affinity assay

HT PagP (10 μM) was bound to Ni-Sepharose (GE Healthcare) resin in 6 M GuHCl, 250 mM NaCl, and 10 mM Tris, pH 8.0, containing 5 mM imidazole for 2 h at 4 °C. The resin was then washed with deionised water followed by equilibration in 50 mM glycine, pH 9.5. Ten micromolar lysozyme, SurA or Skp trimers were then added and allowed to bind for 1 h in 50 mM glycine buffer, pH 9.5 containing 5 mM imidazole. The resin was then washed with 20 mM imidazole in 50 mM glycine, pH 9.5. Elution of bound protein was carried out at room temperature using 50 mM glycine, pH 9.5, containing 5 M urea and 500 mM imidazole. The final eluent and the unbound fractions were analysed by SDS-PAGE.

### Kinetic folding experiments

Changes in Trp fluorescence emission measured at 335 nm upon excitation at 280 nm were used to monitor PagP folding using a Photon Technology International Fluorimeter (Ford, West Sussex, UK) equipped with a thermally controlled four-cell changer. The temperature was maintained at 37 °C using a circulating water bath. A stock of 100 μM PagP unfolded in 10 M urea was rapidly diluted into buffer containing liposomes and urea to initiate folding. Samples were mixed manually and contained a final concentration of 0.4 μM PagP, 1.28 mM liposomes (LPR 3200:1), and varying concentrations of urea. Kinetic transients were followed for up to 2 h. For the kinetic experiments in the presence of periplasmic folding factors or lysozyme, PagP was first incubated in the presence of an excess of chaperone or lysozyme (threefold molar excess of SurA or lysozyme or a twofold molar excess of Skp trimers) for 5 min at room temperature in 50 mM glycine buffer, pH 9.5, and 0.24 M urea before being diluted sixfold into 50 mM glycine buffer, pH 9.5, containing liposomes and varying concentrations of urea (2–4 M). Samples contained a final concentration of 0.4 μM PagP and 1.28 mM liposomes (LPR 3200:1). Control experiments were carried out to ensure that this protocol did not alter the observed rate of PagP folding in the absence of Skp or SurA. Kinetic traces were fitted to a single exponential function *y* = *A* ⋅ *e*^− *k*∙ *t*^ + *c*, where *k* is the folding rate constant. For each sample, four or more replicate traces were fitted using the global fit package in Igor Pro 6.0 (Wavemetrics) sharing the rate constants. Each rate was measured using three different batches of liposomes, and the rate constant was obtained from the global fits for each batch averaged. The standard error of the mean was then calculated from the equation $\mathrm{SEM}=\frac{\sigma }{\sqrt{n}}$, where σ is the standard deviation of the rates and *n* is the number of liposome batch replicates.

### SDS-PAGE analysis of folding yields

Samples used in the folding assays had a final PagP concentration of 4 μM and a lipid concentration of 12.8 mM (to give an LPR of 3200:1) in buffer containing the specified concentration of urea. Typically, samples were allowed to refold overnight (16 h) at 37 °C before folding was quenched by the addition of an equal volume of 2 × SDS-PAGE loading buffer [50 mM Tris–HCl, pH 6.8, 2% (w/v) SDS, 0.1% (w/v) bromophenol blue and 10% (v/v) glycerol]. The samples were then immediately loaded onto a Tris–Tricine SDS-PAGE gel either prior to (termed “cold SDS PAGE”) or after heating (95 °C for 5 min). Gels were stained using Instant Blue stain (Expedeon, UK).

### Skp holdase assay

HT PagP (21.6 μM) was incubated in 50 mM glycine, pH 9.5, and 1 M urea in the presence of a twofold molar excess of Skp trimers or a sixfold molar excess of hen egg white lysozyme. After 5 min, the samples were diluted sixfold into *di*C_12:0_PC liposomes at an LPR of 3200:1 and allowed to equilibrate overnight at 37 °C. The supernatant of each sample after centrifugation was analysed by SDS-PAGE (see method above), with and without boiling, and by Western blotting with mouse anti-histidine tag monoclonal antibody (AbD Serotec). Bands were visualised using the chemiluminescence of horseradish peroxidise-conjugated anti-mouse IgM monoclonal antibody (BD Pharmingen). As a control, each protein was incubated and then mixed with liposomes individually. HT PagP (3.6 μM) solubilised in 50 mM glycine, pH 9.5, containing 10 M urea was included on the gels as a loading control in order to estimate the amount of HT PagP rescued from aggregation by Skp (not shown). Densitometry was carried out using GeneTools image analysis software (Syngene) to estimate the intensity of the relevant bands and the fraction rescued was calculated by dividing the intensity of the test band by the intensity of the loading control.
