# Supplementary material for: Dissecting the Effects of Periplasmic Chaperones on the In Vitro Folding of the Outer Membrane Protein PagP
Source: J Mol Biol. 2013 Sep 9;425(17):3178–91. doi: 10.1016/j.jmb.2013.06.017 (PMC3906610; doi:10.1016/j.jmb.2013.06.017)
Supplement: Supplementary file 1 — Supplementary materials [file mmc1.pdf]

Supplementary information

SI Figure 1

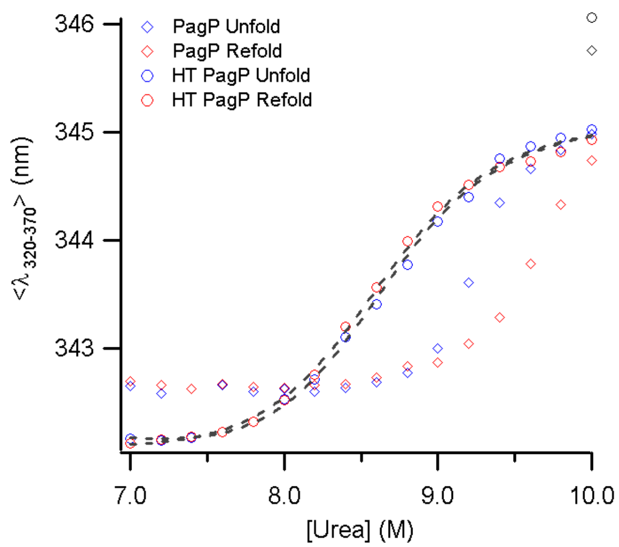

Equilibrium unfolding curves for His-tagged PagP (HT PagP, circles) and untagged PagP (PagP, diamonds). The dark grey dashed lines represent the global fit of the HT PagP curves to a two-state model (described below). The fit parameters obtained ($\Delta G°_{UN}$ = -60.26 ± 6.24 kJ mol^{-1}, M_{UN} = 6.92 ± 0.74 kJ mol^{-1} M^{-1}) are in agreement with those previously measured for HT PagP[1]. The untagged construct did not fold reversibly under these conditions and the data therefore are not fitted. The black symbols indicate the $<\lambda_{320\text{-}370nm}>$ for each PagP construct denatured in 10 M urea in the absence of liposomes. All experiments were carried out at a final protein concentration of 0.4 µM, in *di*$C_{12:0}$PC liposomes, LPR 3200:1, 25 ºC, pH 8.0.

The average wavelength, $<\lambda>$, was calculated according to the following equation:

$$< \lambda > = \frac{\sum_i \lambda_i . I_i}{\sum_i I_i}$$

in which $<\lambda>$ is the average wavelength, λ_i the wavelength and I_i the fluorescence intensity at λ_i. The $<\lambda>$ is calculated over the range i = 320–370 nm.

Since $<\lambda>$ has been shown to vary non-linearly with protein population, the fitting equation used must take into account the quantum yield ratio of the folded and unfolded states of PagP (Q_R)[2; 3]. Q_R was calculated according to the following equation:

$$Q_R = \frac{\Sigma_i \, I_i \, (F)}{\Sigma_i \, I_i \, (U)}$$

where $\Sigma_i \, I_i \, (F)$ is the sum of the intensities of the folded state spectrum over the range of wavelengths used to calculate $< \lambda >$, $\Sigma_i \, I_i \, (U)$ is the sum of the intensities of the unfolded state spectrum over the range of wavelengths used to calculate $< \lambda >$.

This term is then incorporated into the equation for a two-state folding model as previously described[2; 3], and the data fitted using this equation:

$$S_{obs} = \frac{\frac{1}{Q_R}\left[(a[D]+b)\exp\left(\left(\Delta G^{\circ H_2O}_{UN} - M_{UN}[D]\right)/RT\right)\right] + (c[D] + d)}{1 + \frac{1}{Q_R}\left[\exp\left(\left(\Delta G^{\circ H_2O}_{UN} - M_{UN}[D]\right)/RT\right)\right]}$$

where S_{obs} is the observed signal, *a* and *c* are the signals of the native and denatured states, respectively, in the absence of denaturant, *b* and *d* are the denaturant dependence of the signal of the native and denatured states, respectively, *[D]* is the denaturant concentration, $\Delta G^{\circ H_2O}_{UN}$ is the free energy of unfolding in the absence of denaturant, M_{UN} is the m-value (which reflects the denaturant dependence of $\Delta G^{\circ H_2O}_{UN}$) and Q_R is the quantum yield ratio as previously defined.

SI Table 1

Lipid	[Urea] (M)	Rate Constant ($\times 10^{-3}$ s^{-1})	Error in Global Fit ($\times 10^{-5}$ s^{-1})
di$C_{12:0}$PC	2.0	1.37	0.48
	3.0	2.90	1.41
	4.0	7.02	6.22
di$C_{12:0}$PC: *di*$C_{12:0}$PG	2.0	0.32	0.24
	3.0	0.92	0.48
	4.0	1.20	0.49

Urea dependence of PagP folding into *di*$C_{12:0}$PC and 80:20 *di*$C_{12:0}$PC:*di*$C_{12:0}$PG liposomes. Rate constants were obtained by measuring Trp fluorescence emission at 335 nm over time, and fitting the observed transients to a single exponential function (see Methods). Four replicate samples were globally fitted to a single exponential function to obtain the reported rate constants.

SI Figure 2

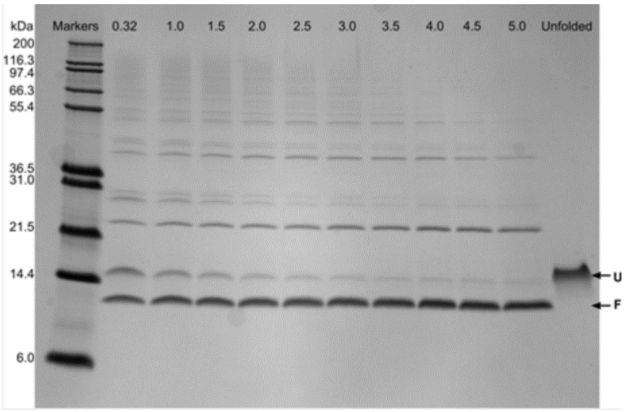

Urea-dependence of PagP folding monitored using cold SDS-PAGE. All samples contained 4 μM PagP (*di*$C_{12:0}$PC liposomes, LPR 3200:1) in 10 mM glycine buffer, pH 9.5, 2 mM EDTA and were refolded at 37 °C for 15 hours prior to 1:1 dilution with 2 × SDS-PAGE loading buffer. Lanes on the

SDS-PAGE gel are numbered to indicate the final concentration of urea (M) in each sample. The unfolded and folded forms of PagP are denoted by U and F, respectively.

SI Table 2

PagP Concentration (μM)	Average Rate Constant ($\times 10^{-3}$ s^{-1})	Standard Deviation ($\times 10^{-4}$ s^{-1})	Standard Error of Mean ($\times 10^{-4}$ s^{-1})
0.4	1.61	7.43	3.71
0.1	2.35	7.98	3.99
0.04	1.56	12.1	6.03

Concentration dependence of PagP folding into *di*$C_{12:0}$PC liposomes. Rate constants were obtained by measuring Trp fluorescence emission at 335 nm over time, and fitting the observed transients to a single exponential function (see Methods). The mean of four transients was calculated, and the standard deviation and standard error (n = 4) were calculated.

SI Figure 3

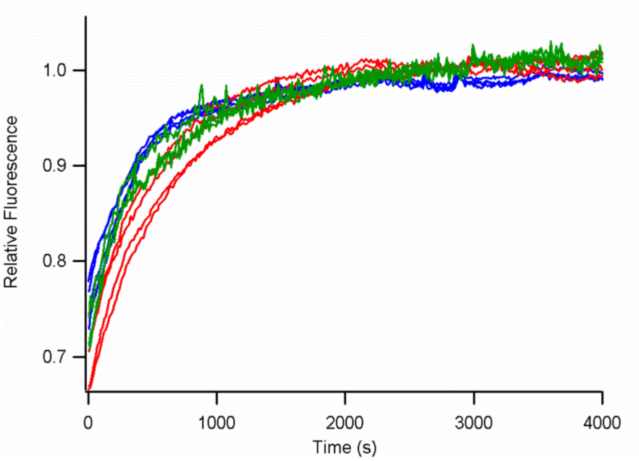

Concentration dependence of PagP folding into *di*$C_{12:0}$PC liposomes. Kinetic traces are normalised to the final fluorescence signal. Samples contained 0.4 μM PagP (red lines), 0.1 μM (blue lines) or 0.04 μM PagP (green lines). All samples contained 50 mM glycine, pH 9.5, 2 M urea and were measured at 37 °C at an LPR of 3200:1. Note that an additional slow phase visible for 0.04 μM PagP was not observed reproducibly in replicate experiments and was thus not considered further.

SI Figure 4

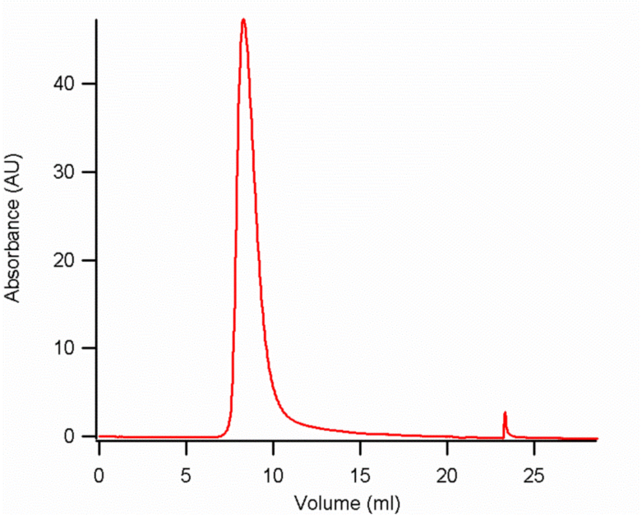

PagP populates a single conformation under the conditions used for chaperone binding. 10 μM PagP in 50 mM glycine, pH 9.5 containing 0.24 M urea was incubated for 5 min at room temperature before injection on to a Superdex 75 10/300 GL column (see Methods).

SI Figure 5

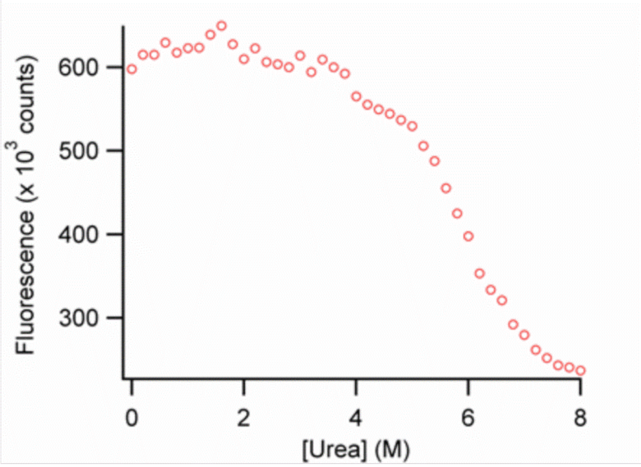

Equilibrium unfolding of SurA monitored using tryptophan fluorescence emission. SurA dissolved in 50 mM glycine buffer, pH 9.5 was diluted into separate aliquots of 50 mM glycine buffer containing different concentrations of urea (0–8 M in 0.2 M increments). The final concentration of protein was 2.5 μM. The aliquots were equilibrated overnight (16 h) at 37 °C before measurement. The fluorescence emission intensity was measured at 335 nm (after excitation at 280 nm) due to the large

intensity difference between the folded and unfolded states at this wavelength, for 60 s and the average signal calculated.

SI Figure 6

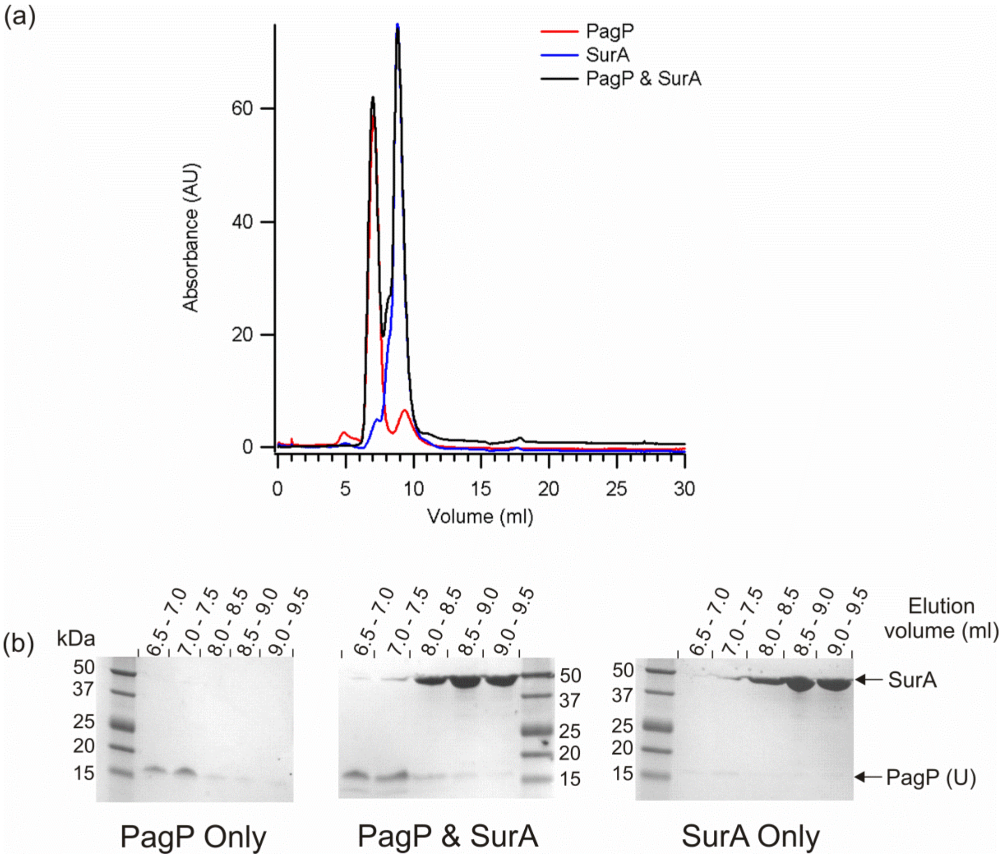

PagP and SurA do not interact under the conditions used in the kinetic folding assays. (a) 10 μM PagP was added to 60 μM SurA in 50 mM glycine, pH 9.5 containing 0.24 M urea and incubated for 5 min at room temperature before injection on to a Superdex 75 10/300 GL column (see Methods). Samples containing either 10 μM PagP or 60 μM SurA in 50 mM glycine, pH 9.5 containing 0.24 M urea were also analysed. (b) Fractions were collected and analysed by SDS-PAGE.

SI Figure 7

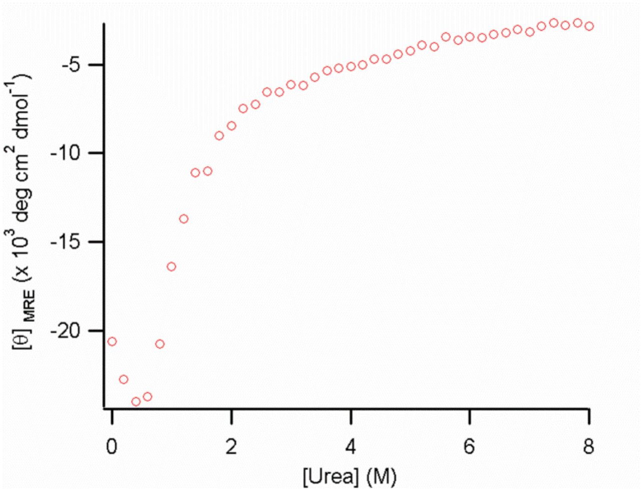

Equilibrium unfolding of Skp monitored using far-UV CD. Skp dissolved in 50 mM glycine buffer, pH 9.5 was diluted into separate aliquots of 50 mM glycine buffer containing different concentrations of urea (0–8 M in 0.2 M increments). The final concentration of protein was 15 µM Skp. The aliquots were equilibrated overnight (16 h) at 37 °C before measurement by far-UV CD spectroscopy. The CD signal of each sample was measured at 222 nm due to the l difference in intensity between the folded and unfolded states at this wavelength, for 60 s and the average signal calculated.

SI Figure 8

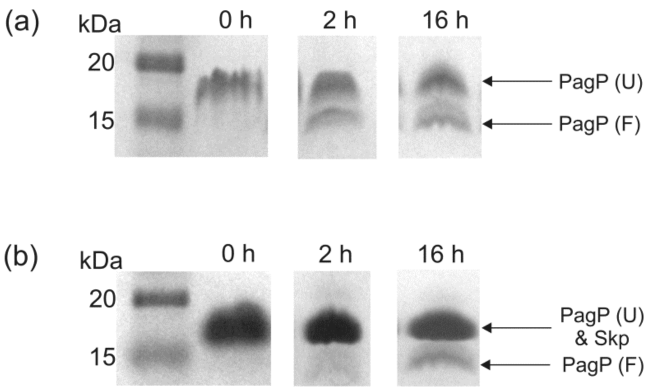

Skp retards the folding of PagP into *di*$C_{12:0}$PC liposomes over a 2 h time course. (a) PagP folding into *di*$C_{12:0}$PC liposomes in the absence of Skp. (b) PagP folding into *di*$C_{12:0}$PC liposomes in the presence of Skp. 12 µM PagP was incubated (with a 2-fold excess of Skp trimers, if appropriate) for 5 min at room temperature in 50 mM glycine, pH 9.5 containing 0.24 M urea before a 3-fold dilution into *di*$C_{12:0}$PC liposomes (LPR 3200:1) in 50 mM glycine, pH 9.5 containing 2 M urea. Samples were removed at 0, 2 and 16 hours and analysed by cold SDS-PAGE immediately.

SI Figure 9

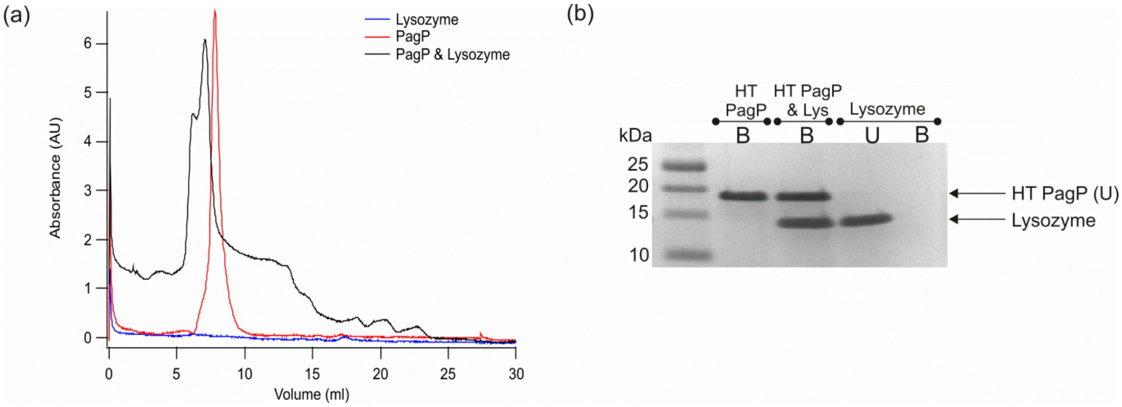

Lysozyme interacts with PagP under the conditions used in the kinetic folding assays as shown by (a) analytical gel filtratration and (b) nickel affinity chromatography. For analytical gel filtration, 2 μM PagP was added to 6 μM lysozyme in 50 mM glycine, pH 9.5 containing 0.24 M urea and incubated for 5 min at room temperature before injection on to a Superdex 75 10/300 GL column (see Methods). Samples containing either 2 μM PagP or 6 μM lysozyme in 50 mM glycine, pH 9.5 containing 0.24 M urea were also analysed. For the nickel affinity assay, 10 μM HT PagP was immobilised on nickel Sepharose resin before incubation with 10 μM lysozyme in 50 mM glycine buffer, pH 9.5. Control experiments containing HT PagP or lysozyme only were conducted under identical conditions for comparison. Lysozyme alone does not bind to the resin and is present in the unbound (U) fraction, while in the presence of HT PagP, lysozyme co-elutes in the bound (B) fraction.

SI Table 3

Lipid	Experiment	Ave Rate Constant ($\times 10^{-3}$ s^{-1})	Std Dev ($\times 10^{-4}$ s^{-1})	Std Error of Mean ($\times 10^{-4}$ s^{-1})
di$C_{12:0}$PC	PagP alone (2M urea)	1.34	0.77	0.45
	PagP & SurA (2M urea)	1.41	2.26	1.31
	PagP & Skp (2M urea)	0	N/A	N/A
	PagP & Lysozyme (2M urea)	0	N/A	N/A
	PagP & NaCl (2M urea)	1.20	2.49	1.44
	PagP, NaCl & Skp (2M urea)	0.81	1.35	0.96
	PagP & LPS (2M urea)	0.89	1.17	0.67
	PagP, LPS & Skp (2M urea)	1.89	5.75	3.32
80:20 *di*$C_{12:0}$PC: *di*$C_{12:0}$PG	PagP alone (3M urea)	0.79	2.00	1.41
	PagP alone (2M urea)	0.28	0.32	0.18
	PagP & SurA (3M urea)	0.63	2.46	1.74
	PagP & Skp (2M urea)	2.41	6.05	4.28
	PagP & Lysozyme (3M urea)	1.52	2.60	1.84
	PagP & NaCl (2M urea)	0.56	1.07	0.62
	PagP, NaCl & Skp (2M urea)	1.53	1.62	0.94
	PagP & LPS (2M urea)	0.68	0.12	0.07
	PagP, LPS & Skp (2M urea)	1.00	2.62	1.51

Measured rates of PagP folding into liposomes *in vitro*. Rate constants were obtained by measuring Trp fluorescence emission at 335 nm over time, and fitting the observed transients to a single exponential function (see Methods). Global fits were obtained over four replicates from a single batch of liposomes, and the average of the global fits from folding reactions into three batches of liposomes calculated. The standard error of the mean was calculated by taking the number of liposome replicates to be 3.

References

1. Huysmans, G. H. M., Baldwin, S. A., Brockwell, D. J. & Radford, S. E. (2010). The transition state for folding of an outer membrane protein. *Proc. Natl Acad. Sci. USA* **107**, 4099-4104.
2. Hong, H. D. & Tamm, L. K. (2004). Elastic coupling of integral membrane protein stability to lipid bilayer forces. *Proc. Natl. Acad. Sci. U. S. A.* **101**, 4065-4070.
3. Moon, C. P., Fleming, K. G., Michael L. Johnson, J. M. H. & Gary, K. A. (2011). Using Tryptophan Fluorescence to Measure the Stability of Membrane Proteins Folded in Liposomes. In *Methods Enzymol*, Vol. 492, pp. 189-211.
